# Intranasal Inoculation of Cryptococcus neoformans in Mice Produces Nasal Infection with Rapid Brain Dissemination

**DOI:** 10.1128/mSphere.00483-19

**Published:** 2019-08-07

**Authors:** Carolina Coelho, Emma Camacho, Antonio Salas, Alexandre Alanio, Arturo Casadevall

**Affiliations:** a W. Harry Feinstone Department of Molecular Microbiology and Immunology, Johns Hopkins University Bloomberg School of Public Health, Baltimore, Maryland, USA; b Medical Research Council Centre for Medical Mycology, Institute of Medical Sciences, University of Aberdeen, Aberdeen, United Kingdom; c Department of Biosciences, College of Life and Environmental Sciences, University of Exeter, Exeter, United Kingdom; d Johns Hopkins School of Medicine, Baltimore, Maryland, USA; e Institut Pasteur, Molecular Mycology Unit, CNRS UMR2000, Université Paris Diderot, Sorbonne Paris Cité, Paris, France; f Laboratoire de Parasitologie-Mycologie, Hôpital Saint-Louis, Groupe Hospitalier Lariboisière, Saint-Louis, Fernand Widal, Assistance Publique-Hôpitaux de Paris (AP-HP), Paris, France; Carnegie Mellon University

**Keywords:** *Cryptococcus*, *Cryptococcus gattii*, *Cryptococcus neoformans*, brain, infection, nose

## Abstract

Cryptococcus neoformans is an important fungal pathogen, causing life-threatening pneumonia and meningoencephalitis. Brain dissemination of C. neoformans is thought to be a consequence of an active infection in the lung which then extravasates to other sites. Brain invasion results from dissemination via either transport by free yeast cells in the bloodstream or Trojan horse transport within mononuclear phagocytes. We assessed brain dissemination in three mouse models of infection: intravenous, intratracheal, and intranasal models. All three modes of infection resulted in dissemination of C. neoformans to the brain in less than 3 h. Further, C. neoformans was detected in the entirety of the upper respiratory tract and the ear canals of mice. In recent years, intranasal infection has become a popular mechanism to induce pulmonary infection because it avoids surgery, but our findings show that instillation of C. neoformans produces cryptococcal nasal infection. These findings imply that immunological studies using intranasal infection should assume that the initial sites of infection of infection are brain, lung, and upper respiratory tract, including the nasal airways.

**IMPORTANCE** Cryptococcus neoformans causes an estimated 181, 000 deaths each year, mostly associated with untreated HIV/AIDS. C. neoformans has a ubiquitous worldwide distribution. Humans become infected from exposure to environmental sources, after which the fungus lays dormant within the human body. Upon AIDS-induced immunosuppression or therapy-induced immunosuppression (required for organ transplant recipients or those suffering from autoimmune disorders), cryptococcal disease reactivates and causes life-threatening meningitis and pneumonia. This study showed that upon contact with the host, C. neoformans can quickly (a few hours) reach the host brain and also colonizes the nose of infected animals. Therefore, this work paves the way to better knowledge of how C. neoformans travels through the host body. Understanding how C. neoformans infects, disseminates, and survives within the host is critically required so that we can prevent infections and the disease caused by this deadly fungus.

## Abstract (precis)

Cryptococcus neoformans causes an estimated 181, 000 deaths each year, mostly associated with untreated HIV/AIDS. C. neoformans has a ubiquitous worldwide distribution. Humans become infected from exposure to environmental sources, after which the fungus lays dormant within the human body. Upon AIDS-induced immunosuppression or therapy-induced immunosuppression (required for organ transplant recipients or those suffering from autoimmune disorders), cryptococcal disease reactivates and causes life-threatening meningitis and pneumonia. This study showed that upon contact with the host, C. neoformans can quickly (a few hours) reach the host brain and also colonizes the nose of infected animals. Therefore, this work paves the way to better knowledge of how C. neoformans travels through the host body. Understanding how C. neoformans infects, disseminates, and survives within the host is critically required so that we can prevent infections and the disease caused by this deadly fungus.

## INTRODUCTION

The genus *Cryptococcus* is populated by environmental fungi, wood-rotting fungi most commonly associated with trees but also with bird guano. Two species of *Cryptococcus* cause disease in humans, characterized mainly by pneumonia and life-threatening meningoencephalitis. Cryptococcus neoformans is the most prevalent pathogen of the genus *Cryptococcus*, causing approximately 200,000 deaths each year, mostly associated with HIV-positive individuals, while the closely related species C. gattii was responsible for an outbreak in British Columbia in apparently immunocompetent individuals (1–3).

The current paradigm for the pathogenesis of human cryptococcosis emerged from a series of observations made over several decades. Exposure is due to inhalation of infectious propagules from environmental niches. Colonization of the lungs by spores, desiccated yeasts, or yeast cells is thought to be quickly controlled by the human immune system via granuloma formation (called cryptococcoma). In the 1950s, silent cryptococcal granulomas were reported in lungs, establishing a parallel to latent tuberculosis (4). In support of this notion, serological studies have established that adults have antibodies to C. neoformans (5) or delayed hypersensitivity skin reactions (6), consistent with asymptomatic infection. Both teenagers and children under the age of 5 living in urban areas are immunoreactive to *Cryptococcus* (7, 8). Finally, HIV patients are infected with a serotype most commonly isolated from the place where they were born or where they spent their infancy (9). Hence, humans are exposed very early in life to these yeasts without developing noticeable disease. The concept of silent or latent infections due to containment in the lungs is further supported by the observation that some recipients of lung transplants develop a cryptococcal infection originating from the donor lung (10–12). Even though most C. neoformans infections in humans are asymptomatic, a significant and unknown proportion of humans become latent carriers of C. neoformans, which may reactivate as host immunity declines.

Given that the initial human infection is thought to start from the lungs, research in the pathogenesis of C. neoformans commonly uses pulmonary infection models. The two inoculation routes most commonly used in mouse models are intratracheal (i.t.) infection and intranasal (i.n.) infection. Intratracheal infection delivers C. neoformans directly to the respiratory tree but requires surgery and significant skill. In contrast, intranasal infection is done by depositing a solution containing C. neoformans in the nose of an anesthetized mouse, which then inhales it, as these animals are obligate nose breathers. Since intranasal infection does not require surgery, it has become a popular mode of induction of C. neoformans infection in mice. Intranasal infection has been accepted as a procedure for inducing pulmonary infection without much investigation as to what actually happens after nose deposition of an infective inoculum. In some cases, researchers use a third route of infection, the intravenous (i.v.) route, since it allows rapid dissemination to the brain, presumably by bypassing the lung immune response (13, 14). Both the intranasal route (13) and the intravenous route (in outbred mice) have been shown to be a relevant model for comparisons to human infection (15–17).

In this study, we compared intravenous, intratracheal, and intranasal infections and observed that all produce rapid brain dissemination. Additionally, we found that intranasal inoculation leads to the presence of yeasts in upper respiratory airways and in the auditory tract. These observations have important implications for the interpretation of intranasal studies in considering immune responses and aspects of pathogenesis.

## RESULTS

**FIG 1:**
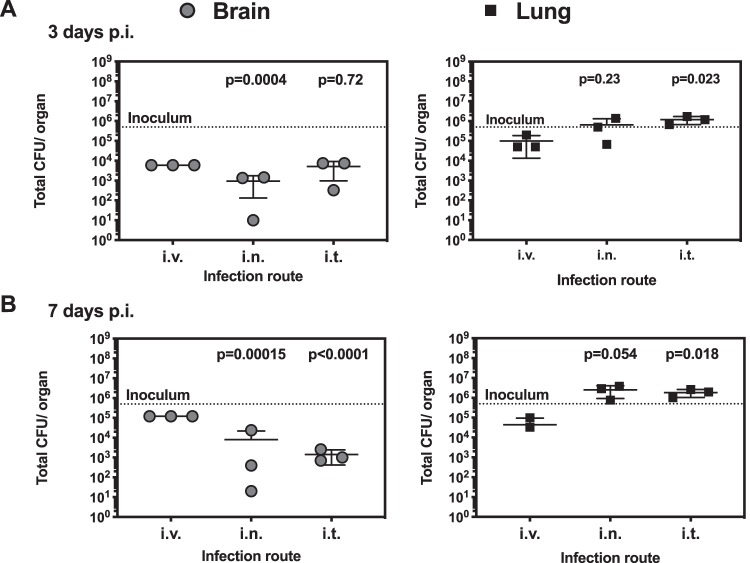
C. neoformans dissemination to the brain and lung after different inoculation routes. Mice were infected with 5 × 10^5^ C. neoformans strain H99E cells via the intravenous (i.v.), intranasal (i.n.), and intratracheal (i.t.) routes. CFU levels in brain (left panels) and lung (right panels) were analyzed at (A) 3 days postinfection (p.i.) and (B) 7 days p.i. Each data point represents one individual mouse, and bars represent means and standard deviations (SD). Numbers represent *P* values calculated from the two-stage linear step-up procedure of Benjamini, Krieger, and Yekutieli, with a false-discovery rate (*q*) of 5% compared to i.v. infection.

We compared 3 routes of infection (intravenous [i.v.], intranasal [i.n.], and intratracheal [i.t.]) to ascertain the kinetics of C. neoformans dissemination to the brain (Fig. 1). We had previously verified that the infectious dose of 5 × 10^5^ CFU induces 100% mortality in 22 days for H99E. We quantified fungal burden in blood, lung, and brains. We detected fungi in blood in two mice, although we also detected bacteria in blood in the same two mice (data not shown). This suggests that the animals with C. neoformans in the blood had severe systemic infection that may have led to concomitant bacterial infection. We conclude that the majority of animals clear C. neoformans from the bloodstream. After 7 days of infection, we measured increased numbers of yeast cells in the brain after i.v. inoculation (more than 10^5^, upper limit of detection) than after i.n. or i.t. inoculation (below 10^4^ CFU). There was a slight increase in fungal burden in the lung when the i.t. route is used compared to the i.v. route. We did not detect differences in brain fungal burden between i.n. inoculation and i.t. inoculation. Remarkably, we noted that there was a substantial amount of yeast cells in mouse brains as early as day 3 after inoculation of yeast by both the i.n. and i.t. routes, which prompted us to ask how quickly C. neoformans disseminates to the mouse brain.

**FIG 2:**
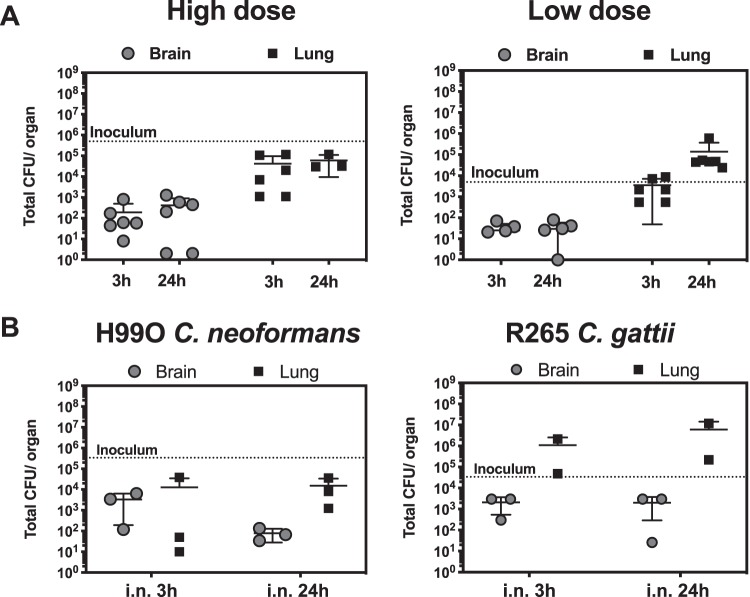
C. neoformans is detected in the brain as early as 3 h postinfection (hpi) after i.n. infection. (A) Mice were infected with either 5 × 10^5^ CFU (High dose) or 5 × 10^3^ CFU (Low dose) of H99E via intranasal instillation and euthanized at the indicated time to measure yeast tissue burden. (B) Mice were infected with strain H99O of C. neoformans or strain R265 of C. gattii. Each data point represents one individual mouse, and bars represent means and SD.

We focused on the intranasal model since this procedure is noninvasive, requiring only a brief period of anesthesia administration and no surgery. We found that the fungal burden in the brain 3 h postinfection with an inoculum of 5 × 10^5^ yeast was on the order of hundreds of CFU for most animals (Fig. 2A). The surprising finding that yeasts were detectable in the brain within hours after i.n. inoculation prompted us to perform additional controls. For one experiment, we rinsed the brains (to remove possible yeast contaminations from the exterior surface of the brain during necropsy), but the results were comparable to those seen in the first experiment (data pooled from the two experiments are shown). We also culled one noninfected (sentinel) mouse at the end of the experiment to measure levels of contamination of tools and materials during necropsy that could have resulted in cross-contamination of the tissue samples. We recovered no CFU from the sentinel mouse, which gave us confidence that the fungal burdens that we detected resulted from brain infection and did not represent accidental contamination (data not shown). To investigate if early dissemination could occur at lower doses, we infected animals with 5 × 10^3^ CFU (at this dose, C. neoformans causes 80% mortality at 40 days). We still observed quick dissemination to the brain, indicating that the fungal burden was not a consequence of exposure to overwhelmingly high numbers of yeasts. Finally, we observed quick dissemination to the brain with strain H99O, a strain closely related to the original H99 clinical isolate of C. neoformans, as well as the R265 strain of C. gattii, the strain associated with the Vancouver outbreak (Fig. 2B). It is possible that the detected yeast burden was arrested in the small capillaries (18–21) or in the postcapillary venules (22) and had not yet invaded the brain parenchyma. However, this is consistent with invasion of the brain, since upon arrest in capillaries, C. neoformans quickly crosses endothelial barriers (19) and possibly the blood-brain barrier. These experiments showed that after intranasal infection, the pathogenic strains of C. neoformans and C. gattii had disseminated to the mouse brain in as little as 3 h.

**FIG 3:**
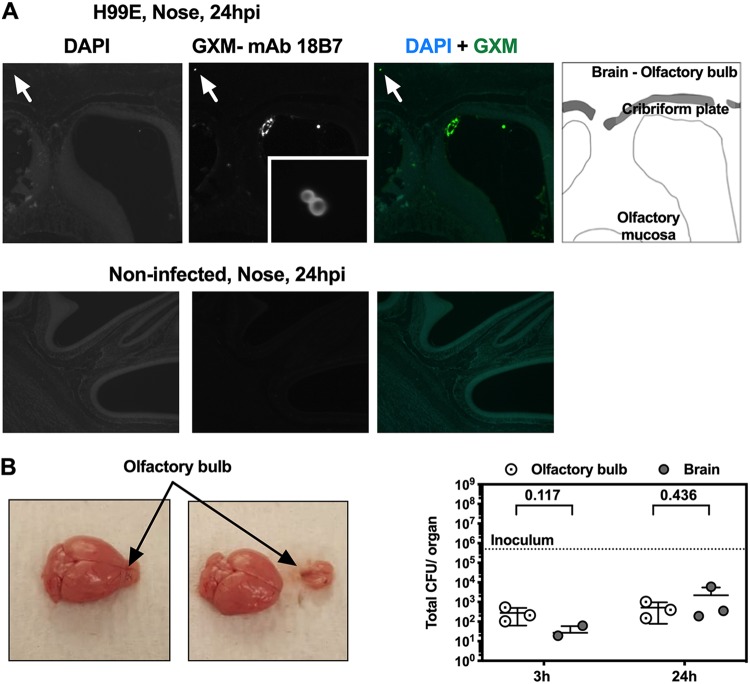
C. neoformans is detected in the brain and the olfactory bulb as early as 3 h postinfection (hpi) after i.n. infection. (A) Mice were infected with 5 × 10^5^ CFU of C. neoformans via intranasal instillation, euthanized at the indicated time, and processed for histology. Yeast cells could be observed in the olfactory bulb of the brain of mice at 24 hpi (top panel, arrows). Noninfected animals were processed under the same conditions to show specificity of staining of yeast capsular immunofluorescence (bottom panel). (B) Mice were infected with high dose of C. neoformans. At the indicated time postinfection, the olfactory bulb was separated from the remainder of the brain (illustrated in the right panel) and CFU were counted (left panel). Each data point represents one individual mouse, and bars represent means and SD. Numbers represent *P* values calculated from two-stage linear step-up procedure of Benjamini, Krieger and Yekutieli, with *q* = 5%.

To confirm the presence of yeast in the brain, we performed immunofluorescence in skulls of infected mice, using a monoclonal antibody against C. neoformans capsular GXM (23). In agreement with the results of CFU quantification, we detected yeast cells (positive immunostaining results and typical morphology) in the mouse brain at 24 h postinfection (Fig. 3A, inset). We wondered if it were possible for C. neoformans to cause damage to the nose mucosa to access the brain, conceivably by accessing the host bloodstream or, alternatively, by using the olfactory nerves to traverse the cribriform plate (as shown for bacterial pathogens [24, 25]) or if after damage the mucosa access the host bloodstream for dissemination. We reasoned that if dissemination occurred through the olfactory system, then the olfactory bulb would contain the majority of yeasts compared to the remainder of the brain. We infected mice and separated their olfactory bulb from the remainder of the brain (Fig. 3B). We found that the olfactory bulb contained amounts of yeasts similar to those present in the remainder of the brain, despite its small size compared to the rest of the brain.

**FIG 4:**
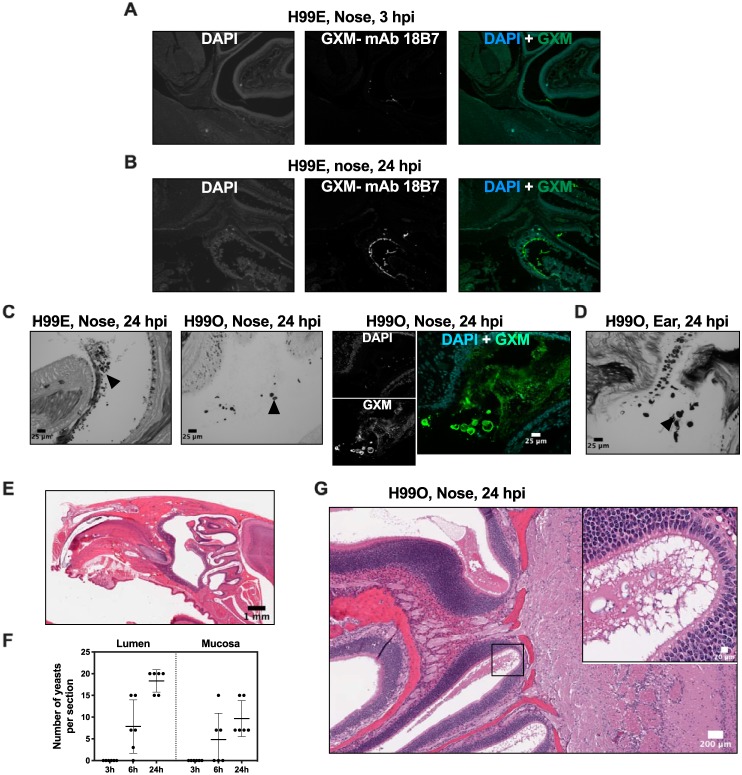
Histopathology of mouse nasal cavities upon C. neoformans infection. Colonization and secretion of GXM in airways of mice was examined. Enlarged yeasts could be observed in airways and in ears. Mice were infected with 5 × 10^5^ CFU of strain H99E or strain H99O (as indicated) of C. neoformans via intranasal instillation, euthanized at the indicated time, and processed for histology. Immunofluorescence staining of medial sagittal cuts of mouse skulls was performed after staining with DAPI (4′,6-diamidino-2-phenylindole) nuclear counterstain and anti-GXM-monoclonal antibody 18B7. (A) Aspects of the cribriform plate and the nose turbinates showing yeasts in the airway at 3 h postinfection. (B) Accumulation of GXM in the nose mucosa at 24 h postinfection. Specificity of staining was confirmed by performing immunostaining in a noninfected mouse (not shown). (C) Grocott methenamine silver staining results for H99E (first panel) and H99O (second panel) and immunofluorescence results for H99O (stacked third and fourth panels and fifth panel) showing enlarged yeast cells in nose airways (arrowheads). (D) Grocott methenamine silver staining showing abundant yeasts and enlarged yeast cells in auditory canal (arrowhead). Bars, 25 μm. (E) Hematoxylin-eosin staining of entire skull (3 h postinfection). (F) Quantification of yeasts in airways (Lumen) and yeasts in nose mucosa. Yeasts were scored via observation from three histological levels from infected mice. (G) Aspect of nose mucosa, close to cribriform plate (24 h postinfection), displaying lack of inflammation and enlarged yeast cells (inset).

To investigate the interaction of C. neoformans with the nose, and possible damage to the nose mucosa that could facilitate dissemination, we performed histological studies using a combination of histology, immunofluorescence, and Grocott methenamine silver and mucicarmine staining (Fig. 4). We found yeasts scattered throughout the upper respiratory tract, particularly in the turbinates of the nose, and some yeasts close to the cribriform plate (Fig. 4A to C), as well as in the ear canals of mice (Fig. 4D). Yeasts were abundant in airways, surrounded with a material that likely represented mucus secretions from the host but that stained abundantly with capsular GXM antibody, indicating a component of secreted polysaccharide in the airways (Fig. 4B). Budding forms rested on the respiratory epithelium cilia and the olfactory epithelium layer, yeast numbers increased from 3 h to 24 h postinfection, and immunostaining of GXM increased (Fig. 4F). We noted that at 24 h postinfection there were rare enlarged yeast cells within the nose turbinates and ears (Fig. 4C to G) whose cell body diameter was above 10 μm and that can therefore be considered titan cells (26–28). The finding of titan cells in the nose of mice is concordant with results reported previously by Lima and Vital (29), who found enlarged cells in noses of guinea pigs after some days of infection. Histopathology analysis detected no damage to the host epithelial layers as well as no inflammatory infiltrate from 3 h to 24 h postinfection; it seems that either yeasts go undetected or they do not cause enough tissue damage to trigger inflammation in the murine host in the early stages of infection. We conclude that, up to 24 h postinfection, the presence of C. neoformans in nose of mice results in minimal (if any) damage to nose mucosa and that the nose shows no sign of inflammation at this stage of infection.

## DISCUSSION

Animal models of infection are critical tools for understanding the pathogenesis of infectious diseases. In the cryptococcal field, mice represent the mammalian species most commonly used to study pathogenesis and immune responses. We compared the kinetics of brain dissemination in the three models of cryptococcal infection that are commonly used, namely, i.v., i.n. and i.t. We were particularly interested in i.n. infection since this approach has become increasingly popular in the field. We found early dissemination to the brain and colonization of upper respiratory tract by C. neoformans and C. gattii. Our data suggest early invasion of the brain and colonization of the nose by cryptococcal species in mouse models.

The quick dissemination to the brain observed in our mouse model is compatible with an hematogenous route of dissemination. Recent work has proposed that escape from the lungs occurs due to phagocyte drainage through the lymphatic system (30). It has not yet been shown how C. neoformans escapes from the lymph nodes into the bloodstream (15, 16, 31), but this will likely be elucidated in the future. We detected no yeasts in the circulating blood of mice, which raises the issue of how the bloodstream is so quickly invaded and quickly cleared. Other groups observed that in i.v. infection, fungi are cleared from the bloodstream within hours or by the day after infection (22, 31), with fungemia resurfacing only very late in the infection. Those observations, together with ours, imply that yeast transit time in blood is very short (20). The short transit time is seemingly due to arrest of yeasts within small capillaries, as can occur in the ears of mice (19) and in the lumen of leptomeningeal capillaries (19, 22). Indeed, our experiments would detect yeast arrested in brain capillaries as yeasts infecting the brain. In contrast with observations detecting a short transit time, others found that blood contains both free yeast cells and yeast cells in the buffy coat, i.e., engulfed by phagocytic immune cells, for up to 20 days postinoculation. Depletion of mononuclear phagocytes decreases the number of yeasts detected in the brain since it prevents Trojan-horse transport (18, 21, 30, 31). Regardless of the duration of transit time, there is sufficient experimental evidence to support the idea that dissemination to the brain occurs via the hematogenous route, by direct crossing of the blood-brain barrier by free yeast cells, and by indirect carriage within host mononuclear phagocytes (Trojan horse transport).

In models of intravenous (i.v.) inoculation, yeast dissemination to the brain occurs as early as 90 min after inoculation (15, 32). The speed of C. neoformans dissemination to the brain was attributed to the intravenous inoculation model, which can simulate fungemia, an event that occurs very late in natural infections. Our findings confirm that i.v. infection disseminated more efficiently to the brain but that all of the infection routes lad to the presence of viable yeasts within the brain as early as 3 h postinfection. Others detected yeasts in the brain as early as 3 days after intranasal infection (33). We did not experimentally confirm that the same quick dissemination to the brain occurs with intratracheal infection, but we expect the intratracheal route of infection to be similar to the intranasal route with respect to the rate of dissemination for 2 reasons: (i) at day 3, the fungal burdens were similar for the two routes; (ii) due to sneezing (and possibly coughing reflexes), inoculation into the trachea would quickly spread C. neoformans throughout the upper respiratory tract. It is our view that once the lungs are infected, the entire upper respiratory tract becomes infected. Therefore, we believe that brain dissemination occurs very rapidly and possibly in a matter of hours, irrespective of the infection route used. This finding and related findings reported previously by others (30, 33) establish that cryptococcal brain dissemination occurs early (in a matter of few hours) and simultaneously with lung infection.

After noting the early dissemination after intranasal inoculation, we wondered if a route of dissemination through a nasal olfactory system could be used by C. neoformans. Both Burkholderia pseudomallei and Listeria monocytogenes can reach the brain via damaging the olfactory mucosa and travelling upward through the olfactory nerve tracts as well as other cranial nerves (24, 25). Neisseria meningitidis invades mouse brains by damaging the olfactory epithelium and travelling along the olfactory tract, through the cribriform plate, to invade the brain (34). The filamentous fungus *Mucor* invades the facial blood vessels from the sinus to reach the eye, the cranial nerves, and the brain. In the case of C. neoformans, the possibility of brain dissemination through the nose was investigated previously (35). In mice, yeast cells were observed along the olfactory nerve and the meninges starting at day 3 after intranasal instillation (35). Our study differed from that previous study in that we focused on the earliest stages of infection and found no evidence of an upward movement that would result in invasion of the brain. We observed no signs of mucosal damage and no inflammation in the nose cavity, despite the presence of abundant amounts of yeasts and secreted GXM. In interpreting these results, we relied on the terminology of the damage-response framework, which defines infection as the acquisition of the microbe by the host and colonization as a state where the damage resulting from the host-microbe interaction is insufficient to affect homeostasis (36). From this perspective, the lack of visible damage suggests that infection leads merely to nasal colonization and not to overt disease. Given the lack of damage and inflammatory infiltrate, our findings do not support the notion that invasion of nasal structures is involved in dissemination of C. neoformans. Nevertheless, intranasal inoculation results in nasal colonization, where fungal cells may later interact with local immune defenses and potentially affect the development of the immune response.

Animal models have shown that some animals can harbor C. neoformans in nose for extended periods of time. Intranasal instillation resulted in the presence of yeast cells detected in the nasal cavities for periods up to 1 month of both mice and rats (37), and the amounts of yeast cells associated with such infection are large enough to be detected by whole-animal noninvasive imaging as early as 1.5 weeks after infection (33). In immunocompetent laboratory mice infected intranasally, yeasts can be detected up to 90 days after instillation (38), and guinea pigs carried C. neoformans in nose for several weeks (29). Some strains of C. neoformans are rhinotropic, with the onset of nasal lesions occurring very late in the disease in laboratory-infected mice (39). Further, C. neoformans is frequently detected in nose of animals outside the laboratory setting. Asymptomatic nasal carriage of C. neoformans has been reported in cats, dogs, and koalas (40, 41). The proportion of positive-testing animals can reach as high as 95%, as reported for feral cats in Italy (42). C. gattii infections are also quite frequent in animals, with studies performed in the British Columbia region reporting positive nasal swab results in 4% and 7% of the cat and dog populations, respectively (43). In conclusion, there is evidence that C. neoformans can survive and even colonize the upper respiratory tract in some felines and rodents, including mice, a common experimental model. This frequent colonization is associated with disease since cryptococcosis is infrequent in species such as cats (44) but is relatively prevalent in koalas (41). Frequent detection of C. neoformans in wild animals and prolonged detection of C. neoformans in noses of laboratory animals show that C. neoformans (and perhaps other *Cryptococcus* species) colonizes the upper airways of animals.

Serological studies have indicated that humans are exposed to C. neoformans at an early age (7) and that C. neoformans can reside in the lungs in a latent form (9). Nonpathogenic species of *Cryptococcus* spp. were identified in several body sites: healthy scalps (45), in mouths of 20% of the healthy population (46), in skin of children (47), and in breast milk (48). Examination of lung transplant patients or bronchiectasis patients frequently detects the presence of *Cryptococcus* but no pathogenic species (49, 50). Indeed, pathogenic species of *Cryptococcus* spp. are rarely found in microbiome studies. The majority of studies in nose and upper respiratory tract (51–56) reported no *Cryptococcus* sp. isolates from nasal cultures, while one study found rare *Cryptococcus* spp. (and no C. neoformans) by culturing nasal cavity lavages (57). There is one notable exception: high-throughput sequencing identified Cryptococcus neoformans as the most abundant fungal species in the middle meatus in 60% of healthy patients and 90% of chronic rhinosinusitis patients in St. Louis, MO, USA (58). The explanation for this striking exception is unknown and may represent a technical problem. Overall, C. neoformans is rarely recovered from healthy humans. In contrast, C. neoformans is frequently isolated from upper respiratory tract of some felines. This discrepancy is likely due to a combination of increased exposure of animals to environmental reservoirs of C. neoformans and immunological differences in host species. However, this is a surprising finding, since animal disease has so far recapitulated human disease (13, 15, 59).

In summary, our study showed colonization of the upper respiratory tract by C. neoformans in mouse models together with rapid dissemination to the brain, independently of the infection route. The rapid appearance of yeast cells in the different body compartments where they initiate local immune responses suggests caution in associating a particular systemic response with a specific tissue. At the very least, our findings suggest the need to revisit long-held views on cryptococcal pathogenesis in animal models of infection using the most modern cellular and immunological tools.

## MATERIALS AND METHODS

C. neoformans was grown from frozen glycerol stocks on a yeast extract-peptone-dextrose (YPD) plate for 2 days and then cultured overnight at 30°C in YPD broth with shaking. We used a strain from the H99E lineage (available from the Jennifer K. Lodge laboratory, Washington University in St. Louis, St. Louis, MO), the H99O strain, a close relative of the original isolate of H99 (60), and the R265 strain of C. gattii, obtained from the American Type Culture Collection.

C57BL/6J mice, aged 8 to 10 weeks, were obtained from Jackson Laboratories and infected with the indicated 5 × 10^3^ (low dose) or 5 × 10^5^ CFU (high dose) inoculum in a final volume of 40 μl of sterile phosphate-buffered saline (PBS) (61). Intravenous (i.v.) injections were performed by injection of 40 μl into the retroorbital sinus of the animal under isoflurane anesthesia. Intranasal (i.n.) experiments were performed by placing 40 μl of yeast suspension into the mouse nares with the mouse under isoflurane anesthesia (62). Intratracheal (i.t.) infections were performed with the mouse under xylazine-ketamine anesthesia. The neck of the animal was exposed, the trachea was exposed via midline incision, and yeasts were inoculated with a 25-gauge syringe directly into the trachea. The incision was closed with Vetbond (3M, St. Paul, MN, USA). Mice were monitored daily for signs of stress and deterioration of health throughout the experiment. All animal experiments were approved by the Johns Hopkins University IACUC under protocol MO18H152.

To measure fungal burden, mice were euthanized and exsanguinated via terminal retroorbital bleeding and tissues were removed and macerated by passage through a 100-μm-pore-size mesh into sterile PBS. The tissue homogenate was then plated into YPD agar plates, and CFU levels were quantified. For one experiment, to remove possible yeast contaminations from the exterior surface of the brain during necropsy, we rinsed the brains. In one experiment, we included a noninfected (sentinel) mouse to test for accidental contamination of tools and materials during necropsy.

For histological analysis of the skull, the skin, lower jaw, and tongue were removed and the remainder of the skull was fixed in Formacal and processed for routine histology. A sagittal cut was performed through the middle section of the mouse skull, and consecutive 4-μm-thick sections were cut at 40-μm intervals for hematoxylin-eosin, mucicarmine, Grocott methenamine silver, and immunofluorescence staining. For immunofluorescence analyses, 18B7 monoclonal antibody against capsular polysaccharides (23) was added and was then detected with anti-mouse IgG_1_-Alexa 488 conjugate antibody. Sections stained with hematoxylin and eosin were scanned using a slide scanner at the Oncology Tissue Services Core of the School of Medicine, Johns Hopkins University. The remaining sections were imaged using an Olympus AX70 microscope (Olympus America, NY, USA). Image cropping and annotation were performed using ImageJ (63).
